# A Rare Case of Metastases to Paranasal Sinus From Colonic Adenocarcinoma

**DOI:** 10.7759/cureus.14718

**Published:** 2021-04-27

**Authors:** Aaditya Prakash, Amitabh Kumar Upadhyay

**Affiliations:** 1 Radiation Oncology, Tata Main Hospital, Jamshedpur, IND; 2 Medical Oncology, Tata Main Hospital, Jamshedpur, IND

**Keywords:** metastatic colo-rectal cancer, palliative radiation therapy, rare metastases, sino-nasal metastasis

## Abstract

Metastases of malignant tumors to the nasal cavity and paranasal sinuses are very rare. Metastases to these locations are usually solitary and produce similar symptoms to those of a primary sinonasal tumor. Pain, nasal obstruction, and epistaxis are the most common symptoms. Although any malignancy could potentially lead to metastasis to the paranasal sinuses, colo-rectal malignancy metastasizes to this site is rare.

We report a case of metastatic adenocarcinoma of colorectal origin to the paranasal sinuses in a 55-year-old female who was initially diagnosed with adenocarcinoma of the colon with lung and liver metastasis. She subsequently developed metastasis to left ethmoidal and sphenoidal sinuses during treatment. A histologic study of the surgical specimen from the sinonasal cavity demonstrated a tumor identical to the patient’s prior primary tumor of the colon. The sinonasal neoplastic tissue showed marked positivity for carcinoembryonic antigen and expressed cytokeratin 20, which differentiates metastatic colonic adenocarcinoma from primary intestinal-type adenocarcinoma (ITAC). She received palliative radiation therapy but died three months after the diagnosis.

These subsets of patients have a poor prognosis. In the majority of patients, palliative therapy is the only possible treatment option. Nevertheless, whenever possible, surgical excision either alone or combined with radiotherapy may be useful for palliation of symptoms and, rarely, to achieve prolonged survival.

## Introduction

The overall incidence of primary adenocarcinoma of the nasal cavity and paranasal sinuses probably accounts for 10% to 20% of all sinonasal malignancies [1]. Most of these tumors are of salivary gland origin. Some tumors are of rare patterns and have histology similar to those of adenocarcinoma of the colon. Metastases of malignant tumors to the nasal cavity and paranasal sinuses are very rare. Incidences of these metastases are common in the 50 and 60 years age group of men and 60 and 70 years age group of women [2]. A review of literature on tumors of paranasal sinuses reports very few cases of sinonasal metastases from colorectal carcinoma [3-8].

## Case presentation

**Figure 1:**
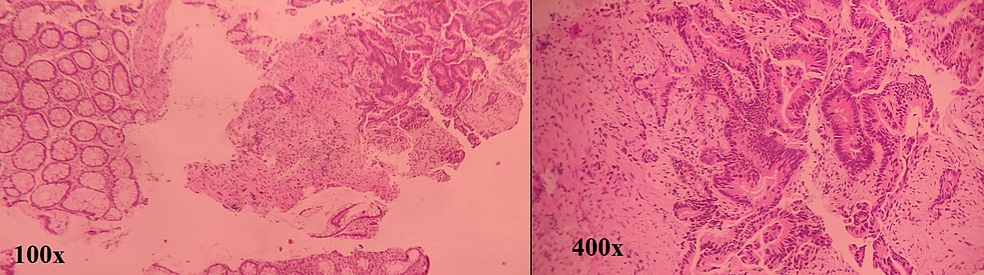
H&E stained colonic endoscopic biopsy sections showing infiltrative, branching glands lined by atypical cells: 100× and 400× H&E: hematoxylin and eosin.

Our patient is a 55-year-old female who presented initially to the general surgery clinic in March 2016 after noticing altered bowel habits for a duration of two months. Her past medical history was associated with type 2 diabetes mellitus and hypertension. She had no previous surgical history, no prior colonoscopies, and no family history of colon cancer. Her only gastrointestinal complaint was the changes in the bowel pattern. There was no history of any melena, hematochezia, or weight loss. A colonoscopy demonstrated a splenic flexure mass obstructing the transverse colon lumen and biopsies of the lesion revealed moderately differentiated adenocarcinoma (Figure 1).

Serum carcino-embryonic antigen (CEA) was markedly elevated at 88.52 ng/mL. Computed tomography (CT) scans of the thorax, abdomen, and pelvis were obtained, showing a mass in the transverse colon and peri-colonic adenopathy with evidence of lung and liver metastatic disease. She received four cycles of systemic chemotherapy with the FOLFOX4 regime. Interval CT scans were suggestive of complete regression of lung metastasis. The residual lesion was seen in liver segments of VI and VII. She underwent an extended right hemicolectomy with end colostomy in September 2016 followed by radiofrequency ablation (RFA) of liver metastatic lesions in October 2016. She completed a further course of FOLFOX4 till November 2016. She was kept on routine follow-up as per National Comprehensive Cancer Network (NCCN) guidelines.

**Figure 2:**
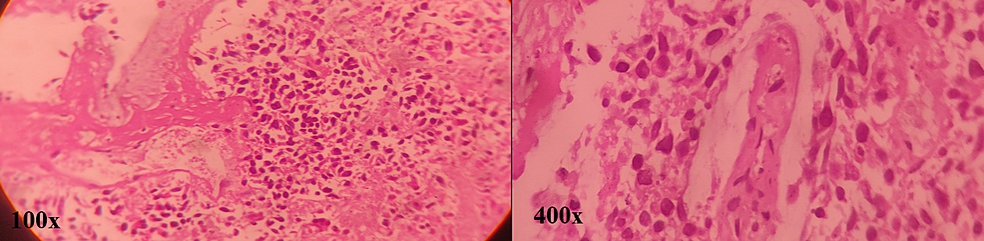
H&E stained section of a fragmented sphenoidal bone biopsy showing scattered metastatic clusters of atypical epithelial cells: 100× and 400× H&E: hematoxylin and eosin.

She underwent a whole-body PET-CT scan as part of a follow-up in June 2017. Findings were suggestive of isolated fluorodeoxyglucose (FDG) avid para-aortic lymph nodes. She underwent exploratory laparotomy with retroperitoneal lymph node dissection (RPLND) and side to side ileo-sigmoid anastomosis in July 2017 followed by 12 cycles of chemotherapy with FOLFIRI regime till May 2018. Her follow-up CT scans of the thorax, abdomen, and pelvis showed an increase in the number and size of pulmonary nodules with an increase in the number of para-aortic lymph nodes. Biopsy from retroperitoneal lymphadenopathy was suggestive of metastatic adenocarcinoma with K-RAS and N-RAS-wild type. She was started on CAPEOX with Bevacizumab-based chemotherapy. In between chemotherapy treatment schedules, she was presented with pain in the left eye with diminution of vision for 15 days. There was an associated left-side facial headache. On eye examination, there was a vision of 6/6 in the right eye and no vision in the left eye. There was a divergent squint in the left eye, at the time of presentation. Contrast-enhanced MRI of the brain and paranasal region revealed mucosal thickening of size 3.2 × 2.6 cm^2^ in the left ethmoidal sinus and sphenoid sinus with extension along the medial wall of the left orbit. Functional endoscopic sinus surgery (FESS) was done. Histopathology reported it as neoplastic etiology (Figure 2).

Immunohistochemistry reported as adenocarcinoma of intestinal type with the positivity of cytokeratin (CK) 20, CDX2, CEA, and negative for TTF1, CK7 immunoprofile. The report was consistent with a colon as primary.

**Figure 3:**
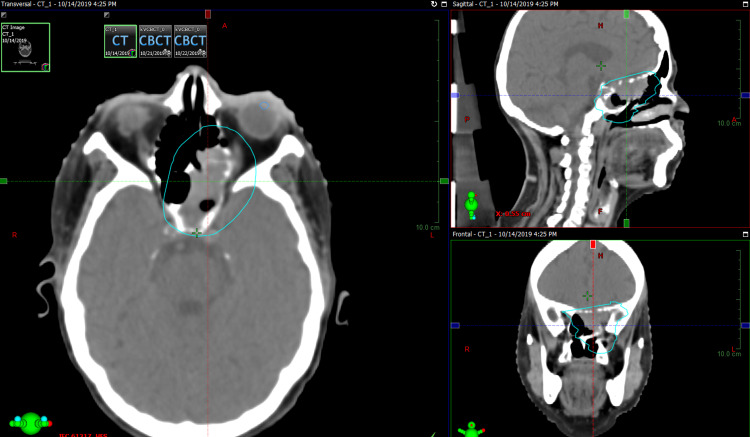
Planning target volume coverage in planning CT scan in all sections (axial, sagittal, coronal; clockwise)

Due to an increase in severity of pain in the left eye and facial region, the patient was planned for palliative radiotherapy to the local site with Intensity Modulated Radiotherapy Technique (IMRT). She received a planning target volume (PTV) dose of 30 Gy in 10 fractions on Varian TrueBeam™ with IMRT (Varian Medical Systems, Inc., Palo Alto, CA) (Figure 3). She tolerated it very well and got significant pain relief at the local site.

She continued on palliative chemotherapy but succumbed subsequently after three months of palliative radiotherapy due to progressive disease and complications.

## Discussion

The most common presentation of symptoms in primary and metastatic malignancy of nasal cavity and paranasal sinuses are facial pain, recur­rent epistaxis, and nasal obstruction [9,10]. It is important to distinguish between primary and metastatic malignancies. Most challenging is to differentiate between histology of colonic metastases to these sites from primary sinus adenocarcinomas, more specifi­cally the colonic variant of intestinal-type adenocarcinoma (ITAC) [11].

Primary ITAC is a rare primary malignancy of the nasal cavity and paranasal sinuses. Generally linked to oc­cupation-related hazard, especially with wood dust expo­sure, but sometimes occurs sporadically [12]. As the histology of the ITAC-colonic variant is diffi­cult to differentiate from metastatic colorectal cancer, therefore, a diagnosis of metastatic adenocarcinoma from these sites requires correlation with any prior history of colorectal cancer. Immunohistochemical studies of the biopsy specimen play an important role to formulate the diagnosis. Positivity for CDX2, CK20, and negativity for CK7 differentiates metastatic colonic adenocarcinoma from ITAC [11,13]. The CK20+/CK7− immunoprofile is considered to be specific for colorectal epithelial tumors [14].

In 1940, Batson postulated that through the low-pressure valveless system connecting deep pelvic veins, intercostal veins, vena cava, and the azygous system, retrograde metastasis to nasal and paranasal sinuses occurs, during increased intrathoracic and abdominal pressure [15].

Nasal and paranasal metastases from colorectal cancer are associated with poor outcomes. None of the cases reports five-year survival from the diagnosis of these me­tastases. All cases were treated with palliative radiotherapy to the nasal and paranasal region [10,11,16]. The mean survival post-palliative radiotherapy was between 2 and 18 months after diagnosis of the metastases [11,16].

## Conclusions

In conclusion, paranasal metastasis is a rare condition and it is important to be differentiated from primary ITAC. The presence of paranasal metastases, unlike ITAC, represents advanced disease and carries a grave prognosis. These types of patients should be managed symptomatically with palliative radiation therapy or chemotherapy with the best supportive care.
